# Single-Cell RNA Sequencing Reveals the Pathogenic Relevance of Intracranial Atherosclerosis in Blood Blister-Like Aneurysms

**DOI:** 10.3389/fimmu.2022.927125

**Published:** 2022-07-08

**Authors:** Dingke Wen, Xing Wang, Ruiqi Chen, Hao Li, Jun Zheng, Wei Fu, Tianjie Zhang, Mu Yang, Chao You, Lu Ma

**Affiliations:** ^1^ Department of Neurosurgery, West China Hospital, Sichuan University, Chengdu, China; ^2^ Radiation Oncology Key Laboratory of Sichuan Province, Sichuan Cancer Hospital and Institute, University of Electronic and Science Technology of China, Chengdu, China

**Keywords:** blood blister-like aneurysm, atherosclerosis, pathogenesis, pathology, single-cell RNA sequencing

## Abstract

**Background:**

Intracranial non-branching site blood blister-like aneurysms (BBA) are extremely rare and vicious. Their etiology remains elusive, and no molecular study has been carried out to reveal its pathogenic relevance to intracranial atherosclerosis. To investigate its transcriptomic landscape and underlying potential pathogenesis, we performed single-cell RNA sequencing with extensive pathological validation.

**Methods:**

In total, 12,245 cells were recovered for single-cell RNA sequencing analysis from 1 BBA and 2 saccular intracranial aneurysms (IAs). Unbiased clustering using Seurat-based pipeline was used for cellular landscape profiling. Cellchat was used to understand intracellular communications. Furthermore, 10 BBAs and 30 IAs were retrospectively collected for pathological validations like scanning electron microscopy, H&E stain, Masson stain, Verhoeff Van Gielson stain, and immunofluorescence.

**Results:**

Single-cell transcriptome profiled 14 total subclusters in 6 major groups, namely, 6 monocyte/macrophage clusters, 2 T&NK clusters, 3 vascular smooth muscle cell (VSMC) clusters, 1 dendritic cell, 1 B cell, and 1 endothelial cell cluster. The only mural cell identified in BBAs was VSMC-2 cluster, while mural cells in IAs comprise most clusters of VSMCs and endothelial cells. Upregulated genes in BBA-derived VSMCs are related to arterial mineralization and atherosclerosis, such as *PTX3*, *SPP1*, *LOX*, *etc.*, whereas vasodilation and physiological regulatory genes such as *MGP*, *ACTA2*, and *MYL9* were conversely enriched in conventional IA-derived VSMCs. Immune cells in the BBA were predominantly macrophages, with a low fraction of T&NK cells, while conventional IAs had a higher percentage of T&NK. Gene enrichment analysis suggested that macrophages in BBA were highly enriched in lipid metabolism as well as atherosclerosis. Ligand–receptor interaction suggested that secretory phosphoprotein 1 (also known as osteopontin) played a major role in mediating the intracellular communication between VSMC and macrophages, especially in BBA. Pathological experiments corroborate with the bioinformatic findings and further characterized BBAs as a thin-walled thrombotic aneurysm with severe atherosclerotic lesions, where ApoE+ macrophages and OPN+ mural cells are intimately involved in the inflammation process.

**Conclusions:**

The preexisting intracranial atherosclerosis might predispose the parent artery to the pathogenic occurrence of BBAs. These data shed light on the pathophysiology of intracranial aneurysms and might assist in the further resolution of the complexity in aneurysm pathogenesis.

## Introduction

More than 85% of human intracranial aneurysms were found in the anterior cerebral circulation and grow at the arterial bifurcations on the circle of Willis (1). They are characterized as chronic pathological bulges protruding from the arterial wall and have mainly been attributed to the structural vulnerability of the branching sites under disrupted cerebral blood flow (2). Consequently, most cerebral aneurysms are termed bifurcating aneurysm or saccular intracranial aneurysm (IAs) based on the berry-like angiographical configurations (2–4). The rest of the intracranial aneurysms that do not exhibit such presentations are classified as arterial dissections or pseudoaneurysms, which are easily distinguishable in practice owing to the representative inflow/outflow conduit or clear preceding trauma history (5–7).

However, some aneurysms do not seem to fall in either category (8). They initiate on the dorsal non-bifurcation sites of the internal carotid artery (ICA) but can still manifest the presentations of a conventional saccular aneurysm (9–16). In 1998, they were first named “blood blister-like aneurysm” (BBA or BBLA) by Ishikawa due to the “red, blister-like” appearance (9). In fact, intracranial non-bifurcation BBAs are extremely rare, accounting for only about 0.3–1% of all intracranial aneurysms, and they have always been challenging for neurosurgeons (11, 17). According to the description by McLaughlin, the imaging presentation can vary into four subtypes in clinical situations, but a common feature they all share is the aberrant occurrence on the non-bifurcated part of the ICA (18, 19). Our previous studies have explored the immune-histological features of BBAs and demonstrated that BBA is not simply a thrombotic dissecting aneurysm but also with large numbers of infiltrated macrophages (16, 20). However, much of the BBA research, including ours, have been descriptive in nature; the etiology of BBA is still an unresolved cerebrovascular enigma (10, 21).

In recent years, single-cell RNA sequencing (scRNA-seq) emerged as an important tool to study the cellular mechanism of arterial disease. It has been used to investigate the transcriptomic features and differentiation landscape at the individual cell transcriptome level on both mural and inflammatory cells under aneurysmal disease (22–27). Previous studies have focused on thoracoabdominal aneurysm secondary to hyperlipidemia, Marfan’s syndrome, or aneurysms at different sections (25–27). By capturing the endothelial cells from endovascular balloons, some pilot investigators evaluated the likelihood of intracranial aneurysm rupture (28). However, no study has yet fully explored the cellular atlas of conventional intracranial aneurysms or some rare subtypes like BBAs.

Therefore, to further explore the molecular pathogenesis of BBA, we performed scRNA-seq on three cases of intracranial aneurysms, including 1 BBA and 2 saccular intracranial aneurysms (IA). Pathological experiments on 10 previous collected BBA and 30 IAs have also been used to validate transcriptomic observations.

## Methods

The data presented in the study are deposited in the National Genomics Data Center, China National Center for Bioinformation Genome Sequencing Achieve (GSA for human) repository, accession number HRA002391. Related data and analytical pipelines will be available from the corresponding authors on reasonable request. All procedures performed involving human participants complied with the ethical standards of the institution (West China Hospital Ethic Committee) and with the 1964 Helsinki Declaration and its later amendments or comparable ethical standards. Patients’ informed and consent forms have been received.

### Human Patient Inclusion

In total, three intracranial aneurysm samples were obtained in this present scRNA-seq analysis, namely, 1 BBA and 2 conventional saccular intracranial aneurysms. For histological validation, additional 10 BBA samples and 30 conventional intracranial aneurysms were included. Patients’ baseline data are shown in **Supplementary Tables S1, S2** (**Supplementary Table S3**). All BBA samples in this present study were initially diagnosed by clinical CT angiography and in-hospital digital subtraction angiography (DSA), fulfilling the diagnostic criteria as follows (1): the patients were confirmed to have had a subarachnoid hemorrhage occurrence (2), the aneurysm grew on the supra-clinoidal segment of the internal carotid artery, (3) the aneurysm showed no relationship with adjacent bifurcating artery (*e*.*g*., ophthalmic artery and posterior communicating artery), and (4) the aneurysm locates on the dorsal or lateral part of the internal carotid artery. Then, the definite diagnosis was finalized based upon intraoperative inspection during craniotomy. The BBA samples were all dissected after satisfactory clipping at the aneurysm neck or additional reinforcement of the parent artery (the detailed procedure was described in previous reports). Similar techniques were used to obtain all other conventional berry-like aneurysms. Their diagnosis also relied on CTA or DSA and a further confirmation in intraoperative view.

### Single-Cell RNA Sequencing and Analysis

The aneurysm samples were obtained and digested in mixed digestion buffer (normal Dulbecco’s modified Eagle’s medium containing 15 mg/ml collagenase type II + 500 ug/ml elastase) for 20–30 mins at 37°C in an incubator. Digested tissue suspension was filtered through a 70-um cell strainer followed by a 40-um strainer. Cells were collected after 600-*g* centrifugation at 4°C. Cell viability and concentration were calculated automatically in a Counting Star system. Cells were then loaded on a chromium controller (10X Genomics) for analysis. A scRNA-seq library was constructed using Chromium Single Cell 3′ v3.1 Reagent Kit as instructed by the official guidelines.

### ScRNA-Seq Data Processing and Visualization

The Single Cell 3′ Protocol-produced library was Illumina-ready; hence, it was sequenced in Illumina system after library construction. Cell Ranger Single-Cell Software Suite was used to process the fastq files exported from sequencing. Cell Ranger uses transcript annotation GTF to bucket the reads into exonic, intronic, and intergenic. Cell Ranger confirmed an exon only if at least 50% of a read is exonic. Cell Ranger was also used in demultiplexing, alignment, filtering, barcode counting, UMI counting, and gene expression estimation for each sample according to the 10X Genomics documentation (https://support.10xgenomics.com/single-cell-gene-expression/software/pipelines/latest/what-is-cell-ranger). Cellranger aggr was used to estimate the gene expression in the samples. Detailed quality control reports of the three samples will be supplied upon further reasonable request.

Downstream analysis using matrix file was conducted in Seurat (version 4.0.5) R package (version 4.1.1) following the standardized analysis pipeline by Satija lab (https://satijalab.org/seurat/articles/pbmc3k_tutorial.html). Each cell with less than 200 genes and more than 25% mitochondrial gene count percentage was excluded from analysis due to low quality. The data set was normalized and logarithmically transformed using NormalizedData and FindVariableFeatures common in Seurat package. Batch effect removal was performed during Cell Ranger sorting protocol. Uniform manifold approximation and projection (UMAP) was used to visualize cell clustering. Unsupervised clustering was conducted by adjusting the resolution index from 0.1 to 1.0 to identify the most appropriate data. Dotplot and Featureplot was used to visualize the enriched expressing genes. Differentially expressed genes were detected by default Wilcoxon test. Differentially expressing genes in smooth muscle cell clusters were detected compared to the rest of the clusters. Differentially expressed genes in macrophages were detected in comparison to other macrophage clusters. Kyoto Encyclopedia of Genes and Genomes (KEGG) analysis was performed by clusterProfiler (version 4.20) to identify potentially enriched cellular pathways. Cellchat package was used to analyze the cell signaling pathway, and webr package (version 0.1.5), ggplot2 was used for data visualization.

### Histopathological Assays

All histopathological experiments were performed using previously collected aneurysm samples. Th detailed inclusion criteria and fixation protocol were described in our previous publications. Briefly, 10 BBA samples and 30 IA samples were included for histopathological validation. In the present study, H&E stain and Verhoeff Van Gielson stain were used to investigate the aneurysm pathology. Scanning electron microscopy (SEM) was used to visualize the tissue surfaces. Anti-human MYH11, COL1 (collagen-1), MGP, and OPN (osteopontin) were used separately to identify the aneurysm tissue component cells. CD3, CD68, CD206, and ApoE were used separately to identify the immune cells in aneurysm. Detailed antibody information was included in **supplementary files** (**Supplementary Table S3**).

### Statistical Analysis

For pathological validations, positive areas fraction and elastic fiber fraction were all calculated by open-source ImageJ (Ver 2.1.0) software. Views were selected from captured figures under a microscope at ×20. Cells that are positive in immunofluorescence stain were manually counted with a cell counter function in ImageJ. For continuous data with equal variance, Student’s *t*-test was used for BBA-IA intergroup comparison. For data with unequal variance, Welch’s test was used (*p-value < 0.05, **p-value < 0.01, ***p-value < 0.001, ****p-value < 0.0001). In differential expression analysis, gene sets with |log_2_FC| >0.75, false detection rate (FDR or *q*-value) <0.1, and *p*-value <0.05 were considered statistically significant. SPSS and Prism Graphpad version 8.0 were used for statistical analyses and graph processing.

## Results

### Single-Cell RNA Sequencing Revealed the Cell Population Differences in BBA and IA

**Figure 1:**
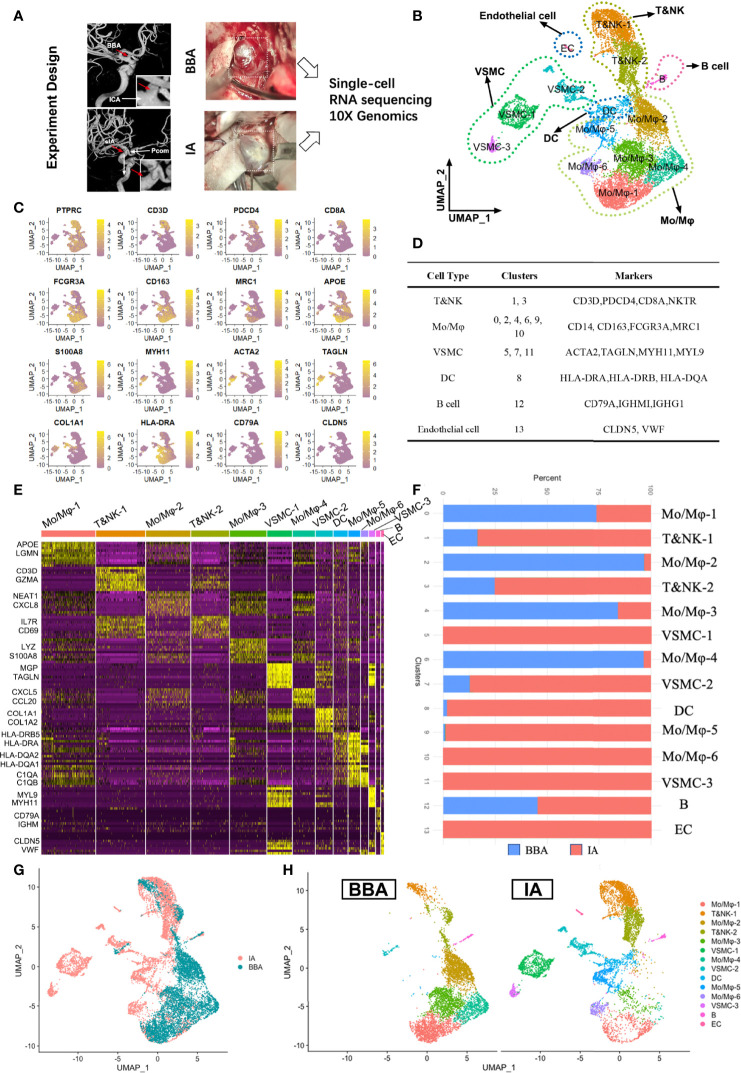
Single-cell RNA sequencing of 2 saccular intracranial aneurysms (IA) and a blood blister-like aneurysm (BBA). **(A)** Experiment design of scRNA-seq. **(B)** Seurat clustering of all cells in aneurysms. **(C)** Featured genes used for classification. **(D)** Major cell types and subclusters of cells. **(E)** Heat map showing the gene enrichment of different clusters. **(F)** Different distributions of cells in BBA and IA. **(G, H)** Distinct distribution of cell clusters in cell atlas.

One BBA and two conventional saccular aneurysms were included in single-cell RNA sequencing (**Figure 1A**). In total, 12,245 cells were recovered for analysis after initial quality control. Moreover, 5,723 cells were obtained from a BBA, while 3,269 and 3,253 cells were collected from two conventional saccular intracranial aneurysms (IAs) separately. Cells from three aneurysms were pooled together for clustering and functional annotation purposes. Unsupervised Seurat clustering identified 14 distinct cell populations in aneurysm, namely, Mo/Mφ-1(*APOE*, *LGMN*, and *SPP1*), Mo/Mφ-2 (*NEAT1*, *CXCL8*, and *CSF3R*), Mo/Mφ-3 (*LYZ, S100A8*, and *VCAN*), Mo/Mφ-4 (*CXCL5*, *CCL20*, and *MACRO*), Mo/Mφ-5 (*HLA-DQA2*, *HLA-DQA1*, and *MRC1*), Mo/Mφ-6 (*C1QA*, *C1QB*, and *CD74*), T&NK cell-1 (*CD3D*, *GNLY*, *NKG7*, and *GZMA*), T&NK cell-2 (*SYNE2*, *IL7R*, *CD69*, and *NKTR*), cells (*HLA-DRB5* and *HLA-DRA*), B cell (*CD79A*, *IGHM*, and *IGHG3*), VSMC-1 (*MGP*, *IGFBP7*, *TAGLN*, and *ACTA2*), VSMC-2 (*COL1A1*, *COL1A2*, and *TIMP1*), VSMC-3 (*MYL9*, *MYH11*, and *TPM2*), and endothelial cells (*CLDN5*, *VWF*, and *RAMP2*) (**Figures 1B, C** and **Supplementary File S1**). In brief, the 14 populations could be simplified into 6 main cell types: T cells and natural killer cells (T&NK-1,2), monocyte and macrophages (Mo/Mφ-1,2,3,4,5,6), vascular smooth muscle cells (VSMC-1,2,3), dendritic cells, B cells, and endothelial cells (EC) (**Figures 1D–F**). Based on the enriched gene features, the BBA and IAs showed distinct cellular distribution trends in cellular components (**Figures 1F–H**). It was obvious that the BBA showed significantly more monocytes/macrophages, especially in Mo/Mφ-1,2,3,4 clusters, but not Mo/Mφ-5,6 or T&NK clusters (**Figure 1F**). Moreover, BBAs have very limited mural cells and barely any ECs; only a very small fraction of VSMC-2 (<12%) subcluster can be identified. B cells, on the other hand, have a similar percentage in both BBA and IA origins (**Figures 1G, H**).

### VSMC Features in BBA and IA

The observation of extremely low mural cells in BBA was largely consistent with previous pathological descriptions on BBAs. However, little was known about their biological process as well as the transcriptomic features. To find out the underlying molecular causes, we set out to investigate the mural cell subpopulation in BBA by comparing to that in the mural cells. Since no EC was not identified in BBAs, we focused on the VSMC clusters.

**Figure 2:**
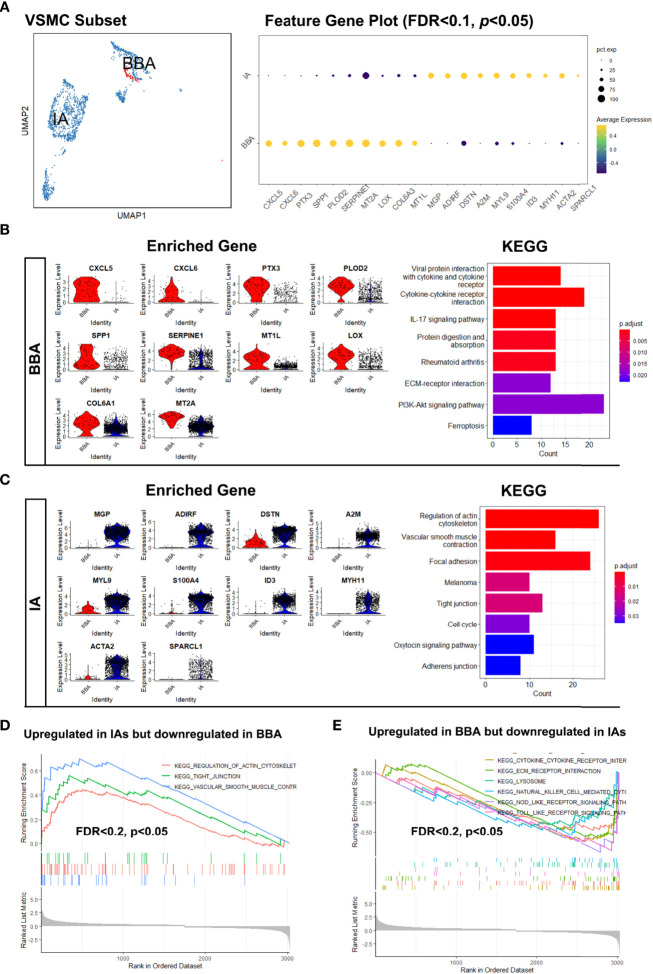
Distinct distribution of vascular smooth muscle cells (VSMCs) in blood blister-like aneurysm (BBA) and intracranial aneurysms (IA). **(A)** The different distributions of VSMCs in uniform manifold approximation and projection and differentially expressed genes. **(B)** Transcriptional features and enriched cellular signaling pathway of BBA-derived VSMCs. **(C)** Transcriptional features and enriched cellular signaling pathway of IA-derived VSMCs. **(D, E)** Upregulated signaling pathways in BBA and IAs profiled in gene set enrichment analysis.

As shown before, the VSMC-1 cluster exhibited a synthetic and proliferative phenotype (*MGP*, *IGFBP7*, and *TAGLN*) while still possessing VSMC feature genes like *ACTA2*. VSMC-2 (*COL1A1*, *COL1A2*, and *TIMP1*) upregulated the genes related to collagen synthesis and chemotaxis, while VSMC-3 (*MYL9*, *MYH11*, and *TPM2*) are physiological contractile types. However, this cannot fully reveal the transcriptional features of VSMCs in BBA. Hence, we re-grouped the VSMCs by their original identity (BBA-derived or IA-derived) (**Figure 2A**). VSMCs from BBAs showed only a small fraction in the UMAP compared to their counterparts in IA. By comparing the enriched gene in VSMCs between IA and BBA, we found that the VSMCs showed significantly discrete gene enrichment patterns. VSMC in BBA-enriched genes like *CXCL5*, *CXCL6*, *PTX3*, *SPP1*, *PLOD2*, *SERPINE1*, *MT2A*, *LOX*, and *COL6A3*. These genes have a high correlation to arterial inflammation, lipid metabolization, severe atherosclerosis, and collagen degradation (|log_2_FC| > 0.75, FDR < 0.1, *p* < 0.05) (**Figure 2B**). *PTX3*, *SPP1*, and *LOX* are important biomarkers for the mineralization and calcification of artery. KEGG analysis also validated the functional predominance in cytokine–cytokine interaction, extra-cellular matrix destruction as well as PI3K signaling pathway activation, whereas for VSMCs in IAs, genes like *MGP*, *ADIRF*, *DSTN*, *A2M*, *MYL9*, *S100A4*, *ID3*, *MYH11*, *ACTA2*, and *SPARCL1* were upregulated. *MGP* and *ADIRF* are key regulators of physiological vascular cell lipid metabolism, while *MYL9*, *MYH11*, and *ACTA2* are responsible for smooth muscle cell contraction and cell–cell conjunction (**Figure 2C**). Further validation *via* gene set enrichment analysis also corroborated the previous observations showing upregulated inflammatory functions and downregulated normal VSMC contraction functions in BBA VSMCs (FDR < 0.2, *p* < 0.05) (**Figures 2D, E**). Such drastic discrepancy in VSMC transcriptomics prompted us that the BBA might be predisposed to more critical intracranial atherosclerotic conditions, where the functions of VSMCs have already been polarized towards a pro-inflammatory phenotype.

### Macrophage-Mediated Innate Inflammation Secondary to BBA and IA

**Figure 3:**
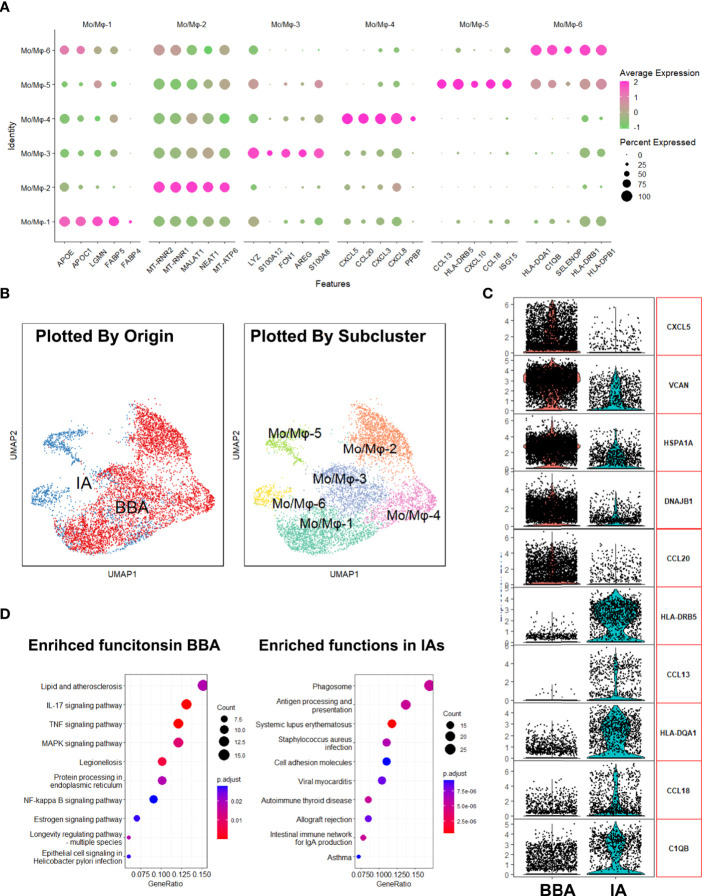
Analysis of macrophages in aneurysms. **(A)** Enriched gene plot of monocyte/macrophage clusters 1 to 6. **(B)** Different distributions of macrophages in uniform manifold approximation and projection grouped by different identities. **(C)** Differentially expressed genes in blood blister-like aneurysm (BBA)-derived macrophages and intracranial aneurysm (IA)-derived macrophages. **(D)** Kyoto Encyclopedia of Genes and Genomes plot showing enriched cellular pathways in macrophages derived from BBA and IAs.

Aiming to investigate the local inflammatory responses in BBA and IA, specialized transcriptional analysis targeting macrophages was thus performed. We first identified the gene signatures of 6 clusters of Mo/Mφ (**Figure 3A**). The feature gene plot suggests Mo/Mφ-1 enriched genes like *APOE*, *APOC1*, *LGMN*, *FABP*, and *FABP4*, which pointed towards an alternative activated phenotype of macrophages. Mo/Mφ-2 enriched genes *MT-RNR2*, *MT-RNR1*, *MALAT1*, *NEAT1*, and *MT-ATP6*, which suggests an inactivated monocyte phenotype. Mo/Mφ-3 exhibited an increased expression of *LYZ*, *S100A12*, *FCN1*, *AREG*, and *S100A8*. These genes indicated a pro-inflammatory phenotype, while Mo/Mφ-4, -5, and -6 are prone to act as chemotaxis and exhibit antigen-presenting functions, which share a somewhat similar function with Mo/Mφ-1 (**Figure 3A**). Then, by grouping the macrophages based on their biological host, we found that the macrophages in BBA showed an apparent dominance in Mo/Mφ-1–4 clusters, while Mo/Mφ-5,6 are exclusively involved in IAs (**Figure 3B**). To understand how the macrophage in BBA differs from that in the IAs, we compared their top 5 highly expressed genes and their upregulated cellular pathways using differentially expressed genes (**Figure 3C**). The analysis showed that *CXCL5*, *VCAN*, *HSPA1A*, *DNAJB1*, and *CCL20* are enriched in BBA-derived Mo/Mφ, while *HLA-DRB5*, *CCL13*, *HLA-DQA1*, *CCL18*, and *C1QB* are enriched in IA-derived Mo/Mφ. KEGG further summarized the BBA-derived Mo/Mφ with upregulating function in lipid and atherosclerosis and IL-17, TNF, AMPK, and NF-kappa B signaling pathways, while IA-derived Mo/Mφ have more abundant cellular pathway functions in phagosome, antigen presenting, and other mild inflammation process (|log_2_FC| > 0.75, FDR < 0.1, *p* < 0.05) (**Figure 3D**). These results provided additional evidence that BBA experienced a more acute and severe arterial damage secondary to atherosclerosis, while IAs show a more moderate and mild pathophysiology.

### The Comparison of Intracellular Communications Between Mural Cells and Inflammatory Cells

Next, we began to interrogate how the mural cells interact with immune cells differently in BBA and IAs. We adopted Cellchat package, which enables us to appreciate the intracellular interaction by predicting the protein–protein interaction using the existing database.

**Figure 4:**
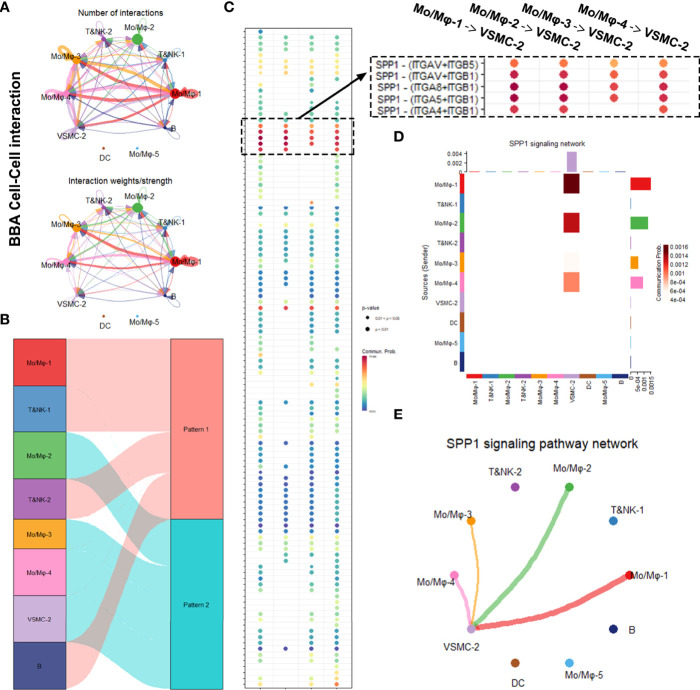
Intracellular analysis between vascular smooth muscle cells (VSMCs) and macrophages in blood blister-like aneurysm (BBA). **(A)** Net plot showing the interaction number and strength. **(B)** River plot showing the communicating and working patterns in cells. **(C)** Network plot showing the specific communicating pathways and their strength. **(D, E)** SPP1 signaling pathways between VSMC subclusters and macrophages in BBA.

We first analyzed the cellular interactions in BBA (**Figure 4**). The net plot showed that Mo/Mφ-1,3,4 radiated the most interactions among all other cell types. VSMC-2, the only remnant mural cells in BBA, received interactions mostly with Mo/Mφ subclusters (**Figure 4A**). Based on shared signaling pathways, the river plot suggested Mo/Mφ-1, T&NK-1,2 as well as B cell worked in synergized inflammatory pathways, while Mo/Mφ-2,3,4 and VSMC-2 acted in another pattern of inflammatory pathways (**Figure 4B**). Detailed cell–cell interaction network showed that the remnant VSMC-2 received signaling pathways *via* various ligand-receptors, among which SPP1- (ITGAV + ITGB5), SPP1- (ITGAV + ITGB1), SPP1- (ITGA8 + ITGB1), SPP1- (ITGA5 + ITGB1), and SPP1- (ITGA4 + ITGB1) signaling pathways were most prominent (**Figure 4C** and **Supplementary File S2**). These cellular activities were most likely contributed by Mo/Mφ-1, followed by Mo/Mφ-2, Mo/Mφ-4, and Mo/Mφ-3 (**Figures 4D, E**).

**Figure 5:**
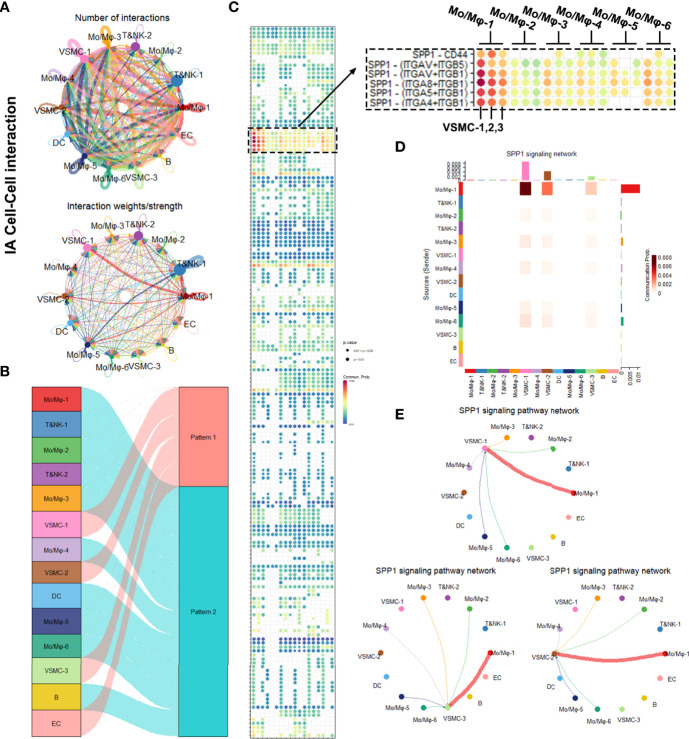
Intracellular analysis between vascular smooth muscle cells (VSMCs) and macrophages in intracranial aneurysms (IAs). **(A)** Net plot showing the interaction number and strength. **(B)** River plot showing the communicating and working patterns in cells. **(C)** Network plot showing the specific communicating pathways and their strength. **(D, E)** SPP1 signaling pathways between VSMC subclusters and macrophages in IAs.

In comparison, a parallel cell–cell interaction analysis was performed on IAs (**Figure 5**). Due to more complexed cell types, the net plot exhibited more complexed intracellular interactions in IAs (**Figures 5A, B**). Notably, the leading communication signaling pathway in IA was still the SPP1 pathways, but with a lower interaction intensity and less coverage in macrophage subgroups (**Figure 5C**). SPP1–CD44 interaction was also exclusively observed in IA rather than in BBA (**Figure 5C**). Furthermore, in conventional IA, SPP1 signaling pathways were still mostly contributed by Mo/Mφ clusters, but Mo/Mφ-2–6 do not seem to contribute in SPP1 signaling pathway compared with that in the BBA (**Figures 5D, E**).

In summary, the SPP1 signaling pathway was significantly upregulated in BBAs, which covers most macrophage subtype and mural cell interactions. IA also upregulated the SPP1 signaling pathway, but with a lower intensity and different ligand-receptor reaction. SPP1 might be a potentially important signaling pathway that underlies the differences between BBAs and IAs.

### Histopathological Validations of the Differences Between IAs and BBAs

**Figure 6:**
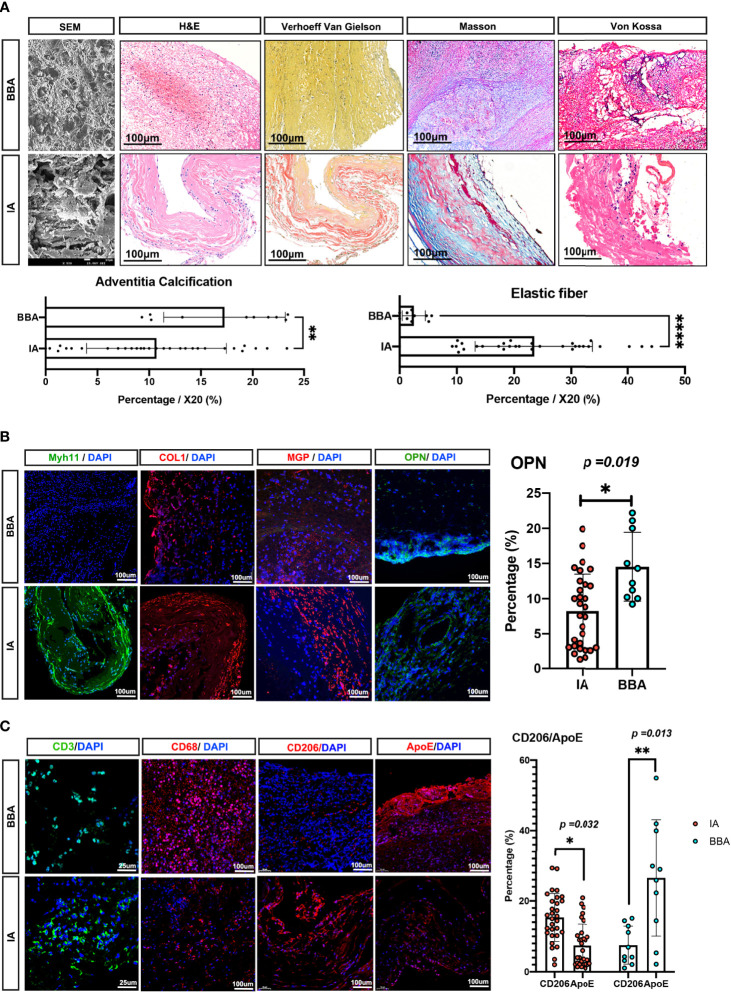
Pathological validations of blood blister-like aneurysm (BBA) and intracranial aneurysm (IA) differences. **(A)** Pathological differences between IAs and BBAs, with statistical analysis. All scale bars are noted in the figures. **(B, C)** Immunofluorescence validation between IAs and BBAs; scale bars are noted in the figures. *Statistically significant; *p*-values were also noted in the plots, *p-value < 0.05, **p-value < 0.01, ***p-value < 0.001, ****p-value < 0.0001.

To provide pathological evidence that the BBAs are secondary to atherosclerotic lesions in intracranial artery, we conducted a pathological comparison in 10 BBA samples and 30 IA samples (**Figure 6**).

SEM first depicted the general appearances of the BBAs, showing the BBA aneurysms manifested as large thrombi with thin atherosclerotic adventitia. In SEM, the cross-section of a BBA aneurysm showed loose connective tissue incarcerated with red blood cells and leukocytes, while IAs exhibit thick collagen bundles and smooth muscle bundles with less infiltrated cells. The H&E stain of BBAs supported the SEM observation, showing no evidence of elastic lamina, with a barely identifiable aneurysm wall, thick mural thrombi, and extremely low mural cells that remained, whereas IAs presented with degenerated vasculature but identifiable aneurysm wall layer. Furthermore, the Verhoeff Van Gielson stain confirmed the absence of elastic fibers, while Masson stain showed a low fraction of collagenous tissue in BBAs (*p* < 0.001). The Von Kossa stain indicated that the BBAs have considerable evidence and traces of calcification tissue on the outer adventitia, whereas IAs have shown less calcification in the aneurysm dome (*p* < 0.01) (**Figure 6A**).

MYH11, COL1 (collagen-1), and MGP were detected to show the characteristics of the mural cells in aneurysms. Apparently, MYH11and MGP are mostly enriched in IA, COL1 was comparable in both aneurysms, whereas OPN was observed to be high in BBAs but low in IAs (**Figure 6B**). To confirm the atherosclerotic inflammation, we noticed a comparable level of CD3+ T cells in both aneurysms and a raised level of CD68+ macrophages in BBAs. ApoE and CD206 (encoded by MRC1 gene, especially enriched in Mo/Mφ-6) were stained separately to identify different macrophages in terms of their distinct cellular functions. It was noted that ApoE+ macrophage in BBA was higher than that in the IAs (BBA *vs*. IA, *p* < 0.013), but not the CD206+ macrophages (IA *vs*. BBA, *p* < 0.032) (**Figure 6C**).

In summary, these results suggested a more severe atherosclerotic pathology in BBAs compared with IAs.

## Discussion

In the present study, we profile the cellular atlas of human intracranial aneurysms from the perspective of single-cell sequencing. The underlying pathogenesis of BBAs was thoroughly investigated in terms of mural cell transcriptomics, inflammatory cell polarizations, and their interacting pathways. Our present study altogether suggested the pathogenic relevance between intracranial atherosclerosis and non-branching site BBAs and further emphasized the significance of SPP1-mediated ligand–receptor interaction in aneurysm progression.

The most inspiring finding of this study is the evidence specifying that severe atherosclerosis predisposes the ICA to BBA occurrence. Initially proposed by Ishikawa, many researchers surmised the traumatic laceration of the internal carotid artery as the direct cause of a BBA (15, 16, 29, 30), but it contradicted against low head trauma rate in BBA patients; the exact pathology that makes ICA susceptible to lacerations remains elusive (16). We speculate that there might be a pre-existing pathological weakness on the parent artery of BBAs. In fact, similar speculations of the BBA etiological relevance to atherosclerosis have been made previously (31–34). However, evidence is lacking due to the inaccessibility and scarcity of samples. As little attention was given to this field, the etiology of BBA is still not known. Currently, single-cell sequencing allows us to carefully solve this myth at the resolution of individual cell level. Therefore, we investigated remnant mural VSMCs in BBA and their functional relevance to atherosclerotic pathology.

VSMCs are the major cellular component in arterial pathology with a high transcriptional plasticity (26, 27, 35). The transcriptome of VSMCs is shaped by vascular inflammatory cues from the microenvironment. Hence, the transcriptome of VSMCs is often used to infer *in situ* arterial pathologies. In BBA-derived VSMCs, several atherosclerotic-related genes are enriched compared with that in the IAs. *CXCL5* and *CXCL6* implicated the acute inflammation mediated by neutrophils and monocytes (36, 37). *PTX*3 is a recognized marker of vascular mineralization and atherosclerosis (38). A high expression of *LOX*, a receptor for oxidized LDL, also suggests that the development of BBAs shared a similar pathogenesis with intracranial arteriosclerosis (39, 40). These results also corroborated with our observation that BBA-derived macrophages show an upregulated expression of *APOE* (encoding apolipoprotein E), an unparalleled anti-atherosclerosis gene (25, 41).

Meanwhile, it is worth mentioning that BBA-derived VSMCs belong to VSMC-2 cluster, a cluster with strong collagen generation and degradation function. VSMC-2 are considered as the synthetic VSMCs or myofibroblasts under pathological condition (42, 43). They are often identified when VSMCs transformed their phenotype due to atherosclerotic plaque formation. The loss of VSMC contractile properties but gaining of fibroblast features enables them to repair vascular injuries, so they are a reckoned hallmark of aneurysmal and atherosclerotic lesions in the central and peripheral arterial systems (43–45). This supports the view by some investigators that the arterial wall is fragile and thin and that only a single layer of fibrous connective tissue plaque is present before the rupture of the aneurysm because the VSMCs have become necrotic or mineralized during the long-term atherosclerotic process, and the residual mural cell component is already very scarce (16, 20, 34, 46).

Another important finding is the prominent role of secreted phosphoprotein 1 (SPP1)

(also known as osteopontin, OPN) and SPP1-associated molecular pathways. Previous studies suggests that OPN fits within the “Goldilocks” paradigm, where the increases in acute phase are vascular protective and calcification attenuative, but their chronic existence correlated with adverse vascular events (47). In intracranial aneurysm studies, SPP1 was not considered a marker of tumor-associated macrophages; SPP1 was instead first described in Shi’s aneurysm genome profile work as a major contributor in extracellular matrix reconstruction and immune cell recruitment (48). Our present study further specified SPP1 as the leading responsible ligand that communicate VSMCs and macrophages, especially in BBAs. This prompts a more activated immune cell activation and recruitment in BBAs. A further study might also personalize the biologic treatment target in aneurysm intervention.

Next, we dissected the transcriptome of macrophages in IAs. We previously provided flow cytometry evidence to show the chronic arterial inflammation process in BBAs. We described an elevated percentage of M2 macrophages in previous flow cytometry studies (20). Nonetheless, the oversimplified classification of M1 and M2 already failed to serve the multifaced research purpose. In this study, we first noticed the overwhelming infiltration of upregulated *APOE* expression in macrophage in BBAs, whereas the mannose receptor, CD206 (encoded by human gene MRC1), was preferentially upregulated in IA-derived macrophages. Previous studies have already noted the discrepancy between CD206 and *APOE* expression, but both are considered resident macrophage markers in tumors and aneurysms alike (25, 41). Hence, the present result might suggest a distinct macrophage function in BBA and conventional IAs. *APOE* might serve as an important biomarker to distinguish BBAs from IAs in molecular pathology.

Last but not the least, we emphasized intracranial atherosclerosis and its pathogenic relevance in BBAs. Intracranial atherosclerosis has gained increasing attention in recent years as a casual cause of ischemic stroke or dementia (49). They can be cryptogenic in non-stenotic cases, which leaves patients neglecting the risk of these insidious lesions (50). Despite the descriptive cohorts in previous decades, mechanism studies regarding the cerebrovascular system remain little (51, 52). Nonetheless, it is already known that they are affected by female hormone. The most common presentation of intracranial atherosclerosis is the fibrous plaque, but rarely complicated pathologies and splitting and laceration most often happen in the fifth decades of the course (49, 53, 54). These conclusions are consistent with the observations in BBAs where the peak of BBA diagnosis happens in female subjects in their 40s–50s and pathologically presented the features of atherosclerosis (12). These results further pointed to a potential research field that connects arterial atherosclerosis to BBA pathogenesis.

In this study, we did not focus on lymphocytes like T, B, and NK cells due to previous disputes regarding their ambiguous roles in aneurysm development (2, 55). However, we did notice a higher percentage of lymphocytes in conventional IAs. In Cellchat analysis, we also described a synergetic effect among T&NK as well as a major subcluster of macrophages. We postulate that this might be the result of co-stimulation required in the activation of innate immunity. Further studies are needed to reveal their molecular importance in aneurysm formation.

This study has certain limitations. Firstly, the samples included in scRNA-seq is still limited. Secondly, the annotation of the cell clusters might be biased by the current disputable biological knowledge. Thirdly, the validation of the present study is limited to immunohistochemistry. Notwithstanding, this study first showed the distinct cellular composition of different intracranial aneurysms by single-cell RNA sequencing and provided new insight into an unsolved pathogenic question of BBA. We also underlie the risk of undiagnosed intracranial atherosclerosis in predisposing non-branching site intracranial aneurysms and proved that scRNA-seq could serve as a powerful tool in studying intracranial aneurysms.

## Conclusions

This study profiled the cellular landscape of intracranial aneurysms, including one blood blister-like aneurysm. It implicated that intracranial arterial atherosclerosis might predispose the parent ICA artery to the pathogenic onset of non-branching site aneurysms. These data shed light on the pathophysiology of various intracranial aneurysms and might assist in the further resolution of the complexity in intracranial aneurysm pathogenesis.

## Data Availability Statement

The data presented in the study are deposited in the National Genomics Data Center, China National Center for Bioinformation Genome Sequencing Achieve (GSA for human) repository, accession number HRA002391.

## Ethics Statement

The studies involving human participants were reviewed and approved by West China Hospital Ethic Committee. The patients/participants provided their written informed consent to participate in this study.

## Author Contributions

DW: drafting/revision of the manuscript for content—including medical writing for content—major role in the acquisition of data, study concept or design, and analysis or interpretation of data. XW: drafting/revision of the manuscript for content—including medical writing for content—and major role in the acquisition of data. HL: major role in the acquisition of data. RC: major role in the acquisition of data. JZ: drafting/revision of the manuscript for content—including medical writing for content—and major role in the acquisition of data. WF: major role in the acquisition of data and analysis of data. TZ: analysis of the data and drafting of the manuscript. MY: major role in the acquisition of data and study concept or design.CY: drafting/revision of the manuscript for content—including medical writing for content—and major role in the acquisition of data. LM: drafting/revision of the manuscript for content—including medical writing for content—major role in the acquisition of data, study concept or design, and analysis or interpretation of data. All authors contributed to the article and approved the submitted version.

## Funding

LM received financial support from the National Key R&D Program of China (numbers 2018YFA 0108604 and 2018YFA0108603) and the Clinical Incubation Program of West China Hospital, SCU (2018HXFU008). MY received financial support from Sichuan International Science and Technology Innovation/Hong Kong, Macao and Taiwan Science and Technology Innovation Cooperation Key Projects (2020YFH0167), Medico-Engineering Cooperation Funds from University of Electronic Science and Technology of China (No.ZYGX2021YGCX005) and Sichuan Cancer Hospital Overseas High-Level Talent Introduction Fund (YBR2019002). ZJ received financial support from the Sichuan Science and Technology Program (2020YFQ0009). WF was supported by the National Natural Science Foundation of China (82101550). The sponsors had no role in the design or conduct of this research.

## Conflict of Interest

The authors declare that the research was conducted in the absence of any commercial or financial relationships that could be construed as a potential conflict of interest.

## Publisher’s Note

All claims expressed in this article are solely those of the authors and do not necessarily represent those of their affiliated organizations, or those of the publisher, the editors and the reviewers. Any product that may be evaluated in this article, or claim that may be made by its manufacturer, is not guaranteed or endorsed by the publisher.

## Supplementary Material

Supplementary File S1 Detailed heat map showing the enriched genes of each cluster annotated in this study. **(A)** Detailed heat map with the top 10 enriched genes. **(B)** Clustering strategies for the present study.

Supplementary File S2 Full cellular interaction network in intracranial aneurysms (IAs) and blood blister-like aneurysm (BBA). **(A)** Cellular interaction pathways in BBA. **(B)** Cellular interaction pathways in IAs.
