# Supplementary figures and images for: Single-Cell RNA Sequencing Reveals the Pathogenic Relevance of Intracranial Atherosclerosis in Blood Blister-Like Aneurysms
Source: Front Immunol. 2022 Jul 8;13:927125. doi: 10.3389/fimmu.2022.927125 (PMC9304558; doi:10.3389/fimmu.2022.927125)

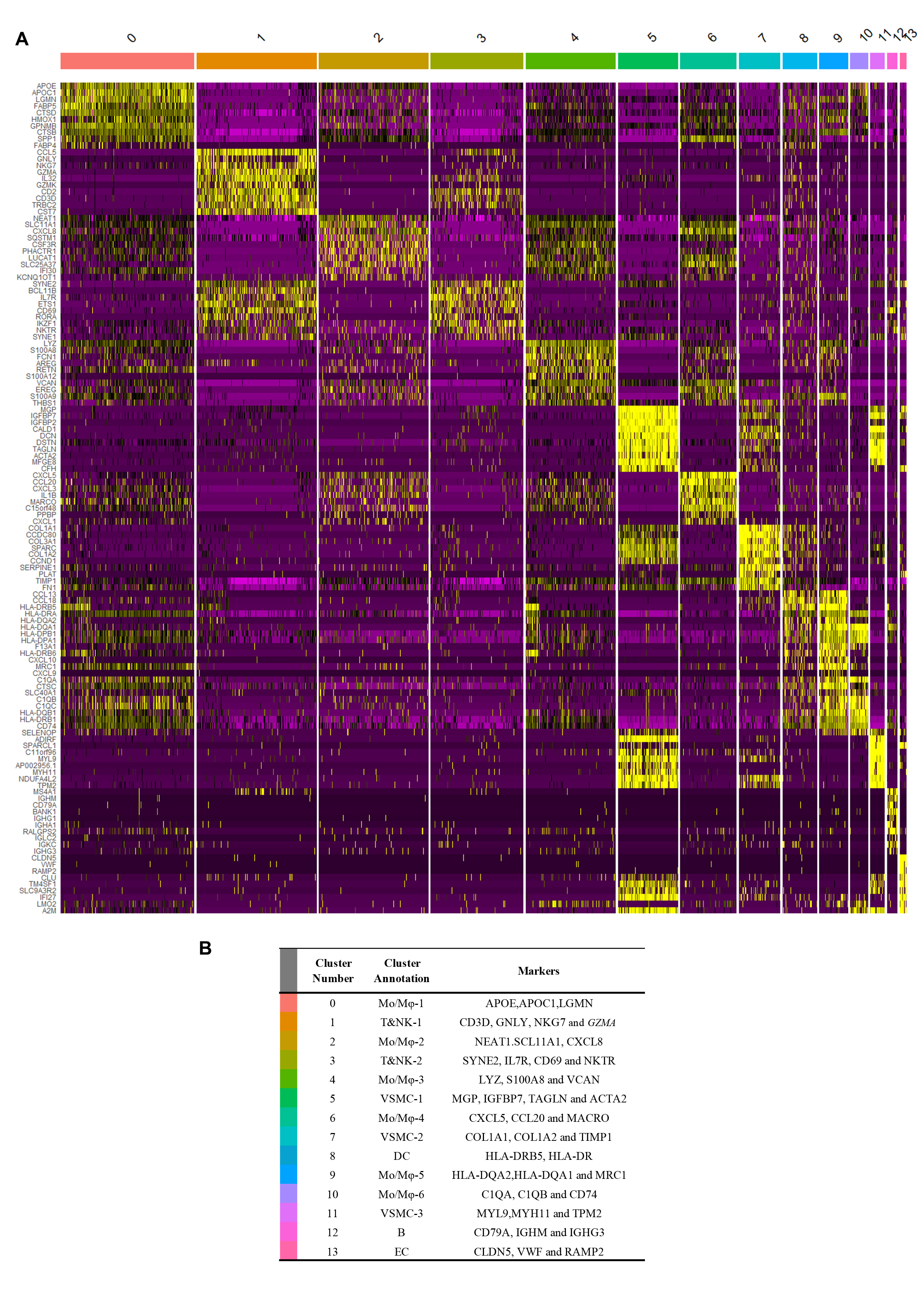

Supplement: Supplementary File S1 — Detailed heat map showing the enriched genes of each cluster annotated in this study. (A) Detailed heat map with the top 10 enriched genes. (B) Clustering strategies for the present study. [file Image_1.jpeg]

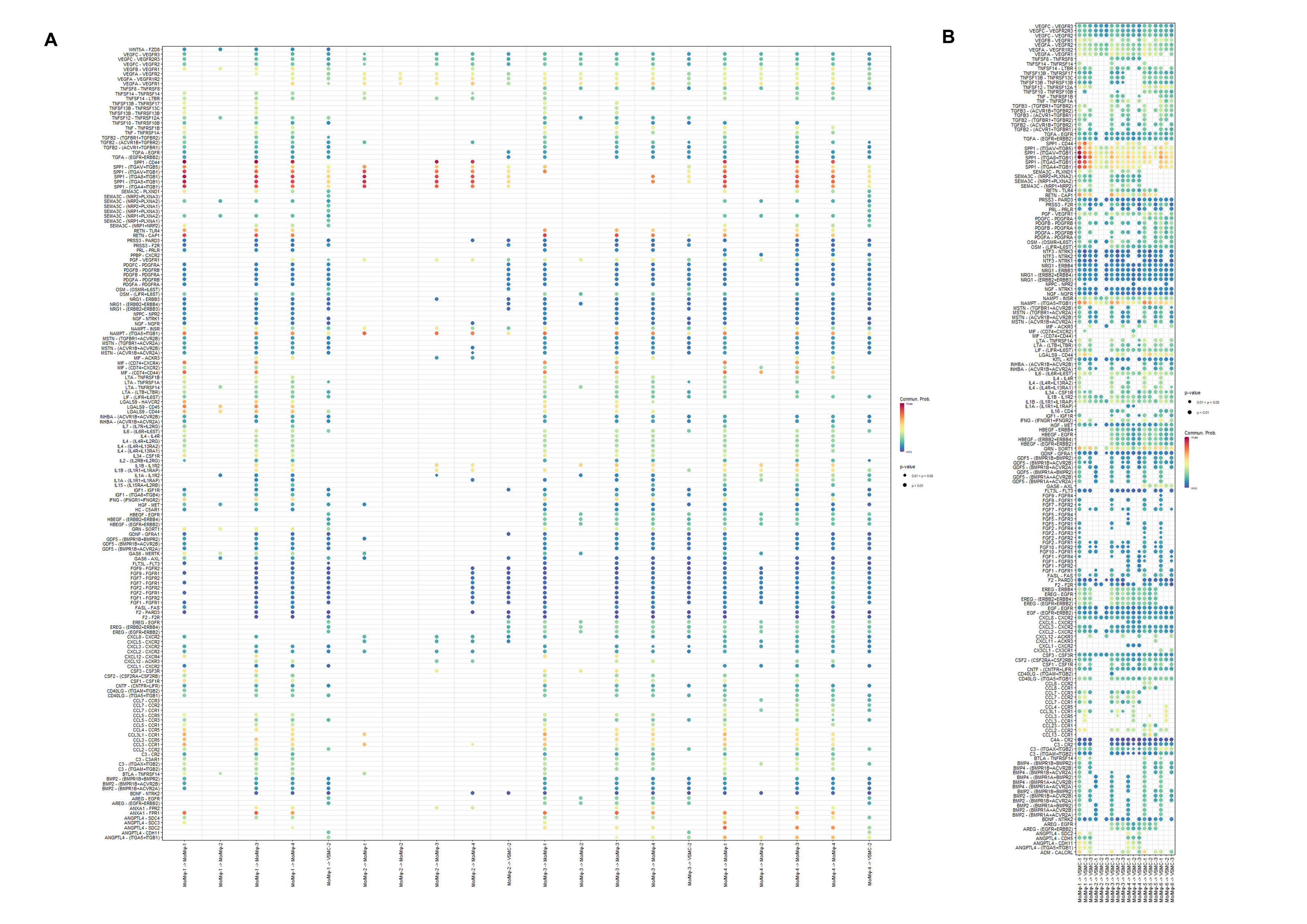

Supplement: Supplementary File S2 — Full cellular interaction network in intracranial aneurysms (IAs) and blood blister-like aneurysm (BBA). (A) Cellular interaction pathways in BBA. (B) Cellular interaction pathways in IAs. [file Image_2.jpeg]
