# Supplementary material for: Single-Cell RNA Sequencing Reveals the Pathogenic Relevance of Intracranial Atherosclerosis in Blood Blister-Like Aneurysms
Source: Front Immunol. 2022 Jul 8;13:927125. doi: 10.3389/fimmu.2022.927125 (PMC9304558; doi:10.3389/fimmu.2022.927125)
Supplement: Supplementary file 3 [file Table_1.docx]

**Supplementary Table 1. Baseline information of saccular aneurysms and blood blister-like aneurysms samples for pathological and immunofluorescence assays.**

**Supplementary Table 2. Baseline information of saccular aneurysms and blood blister-like aneurysms samples used for single cell RNA sequencing.**

Note: GOS Glasgow outcome score.

**Supplementary Table 3. Antibodies used in the assays.**
